# All-inside endoscopic and minimally invasive modified Bunnell suture yield favourable outcomes in acute midsubstance Achilles tendon ruptures: a comparative study

**DOI:** 10.1186/s13018-026-06769-5

**Published:** 2026-03-21

**Authors:** Yue Xue, Nicola Maffulli, Chong Xue, Shun-Hong Gao, Filippo Migliorini, Shi-Ming Feng

**Affiliations:** 1 Sports Medicine Department, Xuzhou Clinical College of Xuzhou Medical University, Xuzhou Central Hospital, No. 199, the Jiefang South Road, Xuzhou, 221009 Jiangsu China; 2 Department of Orthopaedic Surgery, Sant’Andrea Hospital, University La Sapienza, Rome, Italy; 3 Guy Hilton Research Centre, School of Pharmacy and Bioengineering, Keele University, Stoke-on-Trent, Staffordshire, ST4 7QB England; 4 Centre for Sports and Exercise Medicine, Barts and The London School of Medicine and Dentistry, Mile End Hospital, 275 Bancroft Road, London, E1 4DG England; 5 Orthopaedic Department, The Second Hospital of Tangshan, Tangshan, 063000 Hebei China; 6 Department of Trauma and Reconstructive Surgery, University Hospital of Halle, Martin-Luther University Halle-Wittenberg, Ernst-Grube-Street 40, 06097 Halle (Saale), Germany; 7 Department of Orthopaedic and Trauma Surgery, Eifelklinik St.Brigida, 52152 Simmerath, Germany; 8 Department of Life Sciences, Health, and Health Professions, Link Campus University, Via del Casale di San Pio V, Rome, 00165 Italy

**Keywords:** Acute Achilles tendon ruptures, Arthroscopy, Minimally invasive, Suture configuration

## Abstract

**Introduction:**

This study compared the clinical outcomes of the all-inside endoscopic and the minimally invasive modified Bunnell suture configurations for the management of acute midsubstance Achilles tendon ruptures (AMATR).

**Methods:**

A retrospective analysis was conducted on 63 AMATR patients (54 men and 9 women, with a mean age of 39.84 ± 10.40 years (range, 21–62 years). All patients underwent Achilles tendon repair using the modified Bunnell suture configuration using the all-inside endoscopic repair (*n* = 31) or a minimally invasive repair (*n* = 32). The primary endpoint was postoperative functional outcome, assessed using the American Orthopaedic Foot and Ankle Society (AOFAS) score and the Achilles Tendon Total Rupture Score (ATRS) at 6, 12, and 24 months. Secondary endpoints included perioperative and short-term recovery parameters, including operative time, incision length, postoperative pain assessed by the Visual Analog Scale (VAS) on postoperative days 1 and 3, wound complications, and time to return to work and sports activities.

**Results:**

There were no intraoperative complications, and all patients in the endoscopic group achieved primary wound healing. At the 6-, 12-, and 24-month follow-up, both groups demonstrated significant improvement in AOFAS and ATRS scores over time, with no significant differences between groups. Regarding secondary endpoints, the all-inside endoscopic group had a significantly longer operative time but a significantly shorter incision length compared with the minimally invasive group (*p* < 0.05). VAS pain scores on postoperative days 1 and 3 were significantly lower in the endoscopic group (*p* < 0.05). No wound infections occurred in the endoscopic group, whereas three superficial infections were observed in the minimally invasive group; however, the difference was not statistically significant. Patients in the endoscopic group returned to work one week earlier (*p* < 0.05), while the time to return to sports was comparable between groups.

**Conclusion:**

Both the all-inside endoscopic and the minimally invasive modified Bunnell suture configurations provide reliable repair for AMATR and support a successful return to occupational and athletic activity. While the all-inside endoscopic procedure was associated with a longer operative time, it offered advantages in terms of reduced early postoperative pain, smaller incisions, and earlier return to work, without compromising functional recovery at the 2-year follow-up.

## Background

The Achilles tendon is the largest and thickest tendon in the human body. With the recent rapid growth of fitness and recreational sports participation, the incidence of Achilles tendon rupture has increased sharply [1]. Acute midsubstance Achilles tendon rupture (AMATR) occurs most frequently in young and middle-aged men [2], a population typically engaged in high levels of physical activity. Surgical repair offers the advantage of more rapidly restoring tendon function, thereby facilitating an earlier return to work, sports, and daily activities [3–8]. Such outcomes are often less readily achievable with conservative treatment [9].

Traditional open repair, although effective in restoring tendon continuity, typically requires long incisions and is associated with wound-related complication rates of up to 30% [10]. To mitigate these drawbacks, percutaneous and minimally invasive approaches have been increasingly adopted in recent years [11–12]. Minimally invasive procedures have gained increasing popularity for the management of AMATR, largely because they substantially reduce wound-related complications compared with traditional open repair. Recent studies [13–14] have reported a marked decline in postoperative adverse events with minimally invasive approaches, with rates of sural nerve injury and re-rupture reduced to 1.67% and 1.25%, respectively. However, concerns remain regarding the quality of tendon purchase with percutaneous techniques. Biomechanical investigations [15] have shown that only about 55% of the suture material reliably penetrates the tendon substance, potentially weakening the repair construct. Moreover, inadvertent capture of the paratenon may impede tendon gliding and negatively influence healing [16–17]. These limitations have prompted growing interest in endoscopic repair, which enables direct visualization of the ruptured tendon, allowing for more accurate suture placement while maintaining the advantages of a minimally invasive approach [18]. Endoscopy has recently been applied more broadly in the management of tendon injuries, offering a promising refinement of minimally invasive Achilles repair [19]. To date, reported endoscopic Achilles tendon repair techniques mainly include endoscopy-assisted percutaneous repairs and all-inside endoscopic repairs. Endoscopy-assisted approaches reduce sural nerve injury, but still rely on percutaneous stitching and may provide less reliable fixation. All-inside techniques allow direct visualization and secure stitches with no nerve injuries observed. However, evidence on the use of an all-inside endoscopic approach for Achilles tendon repair remains limited [20–21], and comparative data with the minimally invasive technique are still scarce.

The purpose of this retrospective cohort study was to compare surgical time, clinical outcomes, and complications between an all-inside endoscopic modified Bunnell repair and a minimally invasive modified Bunnell repair for AMATR. It was hypothesised that the all-inside endoscopic technique would provide a safe and reliable alternative to the minimally invasive approach, offering evidence to guide surgical decision-making for AMATR.

## Methods

### Patients

The study was approved by the institutional review board. All participants provided written informed consent to participate in the study, and authorization in accordance with the Health Insurance Portability and Accountability Act was also obtained. We retrospectively reviewed patients with AMATR who underwent surgical treatment performed by a single fellowship-trained surgeon at our hospital between March 2019 and February 2023.

### Inclusion and exclusion criteria

Inclusion criteria were as follows: (1) unilateral acute closed Achilles tendon rupture diagnosed within 2 weeks; (2) presence of a palpable gap at the rupture site with increased ankle dorsiflexion; (3) positive Simmonds and Matles tests; (4) confirmation of rupture by ultrasound or magnetic resonance imaging (MRI); and (5) treated with either all-inside endoscopic or the minimally invasive modified Bunnell suture configuration.

Exclusion criteria included: (1) follow-up duration of less than 24 months; (2) prior Achilles tendon rupture or surgery; (3) rupture located within 2 cm of the tendon insertion or at the musculotendinous junction; and (4) systemic metabolic disorders or long-term corticosteroid/other medication use.

Prior to surgery, all patients were informed of the estimated operative time, costs, and expected outcomes. They were then offered a choice between two surgical procedures: (a) all-inside endoscopic modified Bunnell suture configuration repair or (b) minimally invasive modified Bunnell suture configuration repair. For patients unable to make a decision, the choice of procedure was made by tossing a coin.

### Surgical technique

All patients underwent either intravenous or spinal anaesthesia. Following induction, patients were positioned prone with a 10 cm cushion under the operative limb. A pneumatic thigh tourniquet was applied at 300 mmHg without exsanguination. All surgeries were performed by the same senior surgeon.

**Fig. 1:**
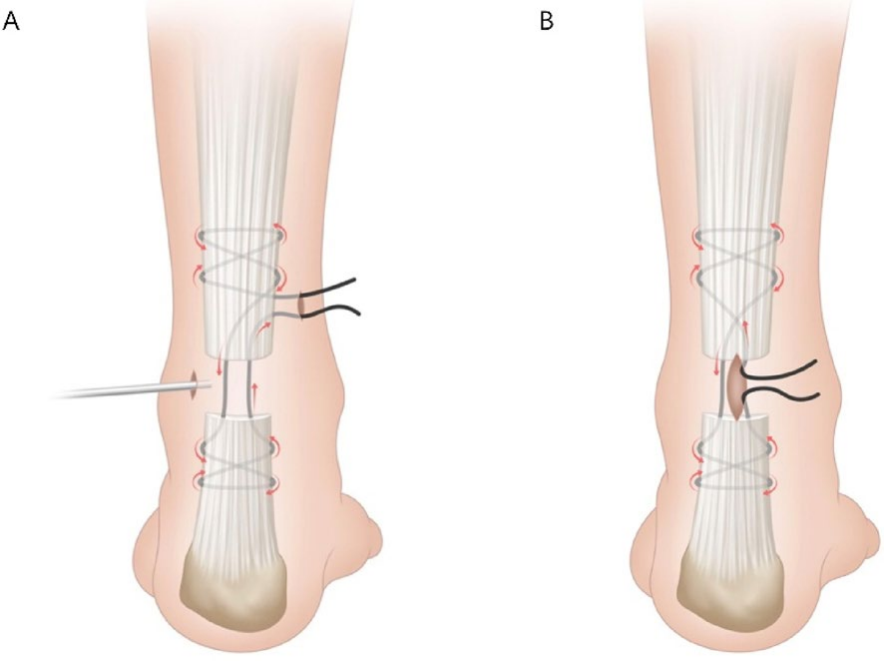
Surgical diagrams of endoscopic **A** and minimally invasive **B** modified Bunnell suture configurations

**Fig. 2:**
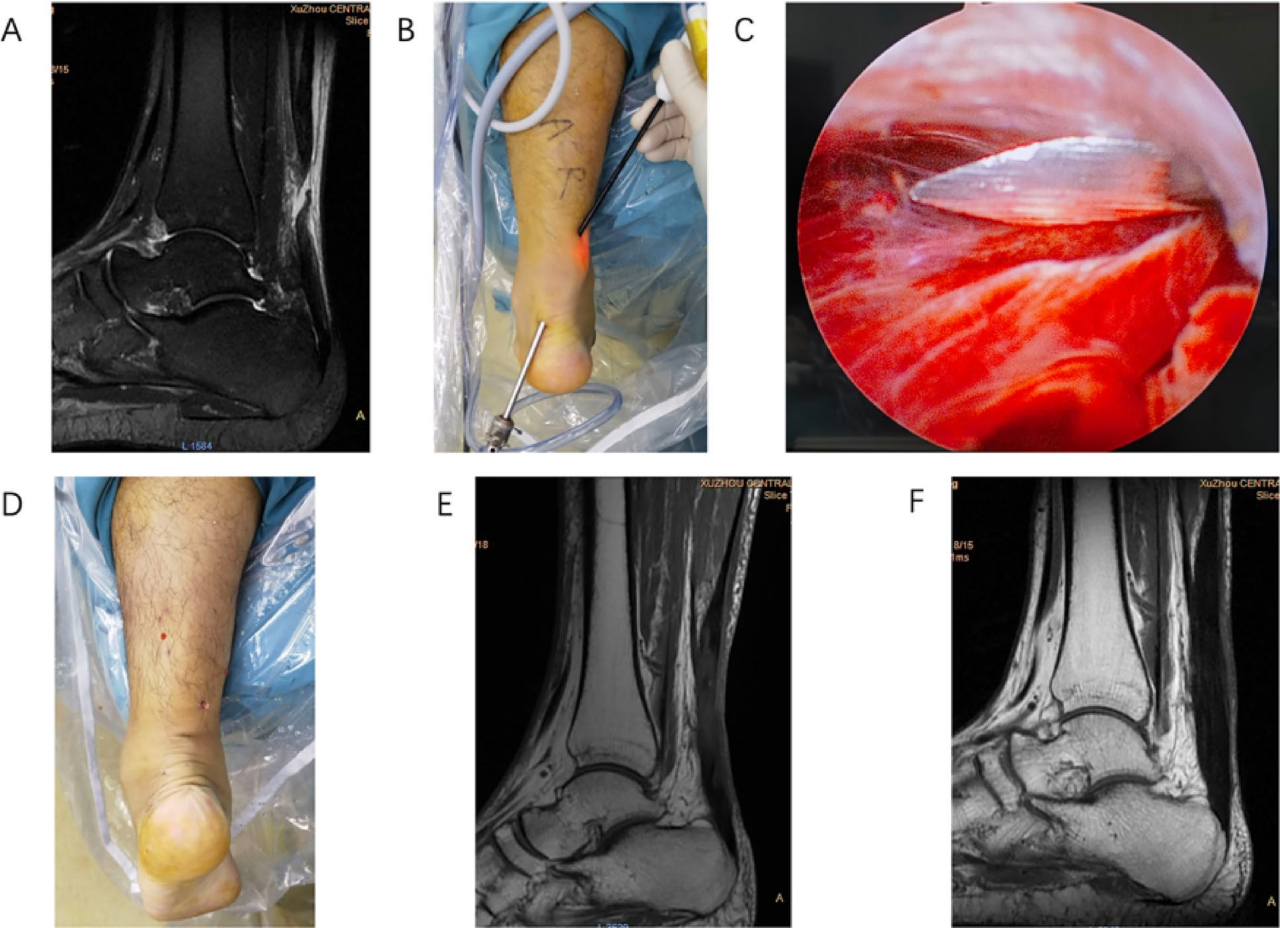
The two-portal all-inside endoscopic modified Bunnell suture configuration to repair an acute midsubstance Achilles tendon rupture on the left side. **A** acute midsubstance Achilles tendon rupture confirmed at magnetic resonance imaging (MRI); **B** overview of the endoscopic portals; **C** endoscopic view of the ruptured Achilles tendon with the suture needle passing through the paratenon; **D** appearance after repair; **E** MRI performed two months postoperatively: satisfactory tendon healing; **F** MRI obtained at six months postoperatively: well-integrated tendon healing

Endoscopic group: After identifying the depression at the site of rupture of the Achilles tendon, a 0.5 cm longitudinal lateral portal was made for insertion of the endoscope. A second 0.5 cm medial portal was then established approximately 3 cm proximal to the rupture site. A high-strength Ultrabrain #2 suture (Smith & Nephew, USA), mounted on a 9 cm Mayo needle or a suture shuttle, was used for tendon repair. Under endoscopic guidance, the needle was passed from the medial portal through the proximal stump and exited laterally, then reintroduced at the same lateral site to produce a lateral-to-medial stitch while avoiding the paratenon. A medial-to-lateral pass was performed to form a Bunnell suture configuration in the proximal stump (Fig. 1A). With the endoscope introduced through the same portal, the identical technique was applied to the distal stump, producing a corresponding Bunnell suture configuration. The tendon stumps were approximated by tightening and securing the sutures with the foot positioned at 20° of plantar flexion. Throughout the procedure, endoscopic visualisation was used to verify that tendon continuity had been restored and promptly identify any technical errors. Successful repair was indirectly indicated when the restored tendon blocked the red glow from the arthroscope light source (Fig. 2).

Minimally invasive group: A 3 to 4 cm longitudinal incision was made over the rupture site. After clot evacuation, the proximal tendon stump was exteriorised with forceps. Using the same suture and needle as in the endoscopic group, percutaneous sutures were placed under palpation, producing a proximal and distal Bunnell suture configuration. Upon completion of the repair, the construct was tensioned. (Fig. 1B). Final tightening of the sutures was performed with the ankle at 20° plantarflexion to optimise tendon apposition and reduce the risk of gap formation.

### Postoperative management and rehabilitation

All patients were managed with an identical rehabilitation protocol. For the first 2 weeks, the ankle was immobilised at 20° plantarflexion using a short-leg brace. From postoperative day 2, active knee and toe motion was encouraged while protected partial weight-bearing with crutches was maintained. Between 3 and 4 weeks, an Achilles walking boot with heel wedges was applied to allow gradual weight-bearing. Dorsiflexion was restricted to the neutral position. At 4 weeks, tendon coaptation was reassessed by ultrasonography, and isometric exercises, isokinetic cycling, and proprioceptive training were initiated. From 5 to 6 weeks, heel wedges were reduced weekly until removal at 6 weeks, and the boot was adjusted to neutral, allowing progression to full weight-bearing and gait training. Active dorsiflexion beyond neutral was permitted at 8 weeks. By 10–12 weeks, strengthening and stretching exercises were introduced, and low-impact activities were resumed. Return to competitive sports was allowed at 6 months postoperatively.

### Follow-up and outcome assessment

Follow-up visits were scheduled every 2 weeks for the first 6 weeks, and every 6 weeks thereafter. The primary endpoint was postoperative functional outcome, assessed using the American Orthopaedic Foot and Ankle Society (AOFAS) ankle-hindfoot score and the Achilles Tendon Total Rupture Score (ATRS) at 6, 12, and 24 months postoperatively. Secondary endpoints included perioperative and short-term recovery parameters, including operative time, incision length, postoperative pain assessed by the Visual Analog Scale (VAS) on postoperative days 1 and 3, wound complications, and time to return to work and sports activities.

### Statistical analysis

Statistical analyses were conducted using SPSS version 26.0. Continuous variables with a normal distribution are presented as mean ± standard deviation. Between-group differences were evaluated using sample t-tests, while repeated-measures ANOVA was applied for longitudinal comparisons. Post hoc analyses were performed with the LSD-t test. A post hoc power analysis was also performed to verify the adequacy of the sample size. The sample sizes of 31 and 32 per group provided less than 10% statistical power for both AOFAS and ATRS to detect a difference in means when analysed using a two-sided two-sample unequal-variance t-test at the 0.05 significance level. Categorical variables were analysed using chi-square tests, and ranked data were assessed with the Mann–Whitney U test. A *p*-value of < 0.05 was considered statistically significant.

## Results

**Fig. 3:**
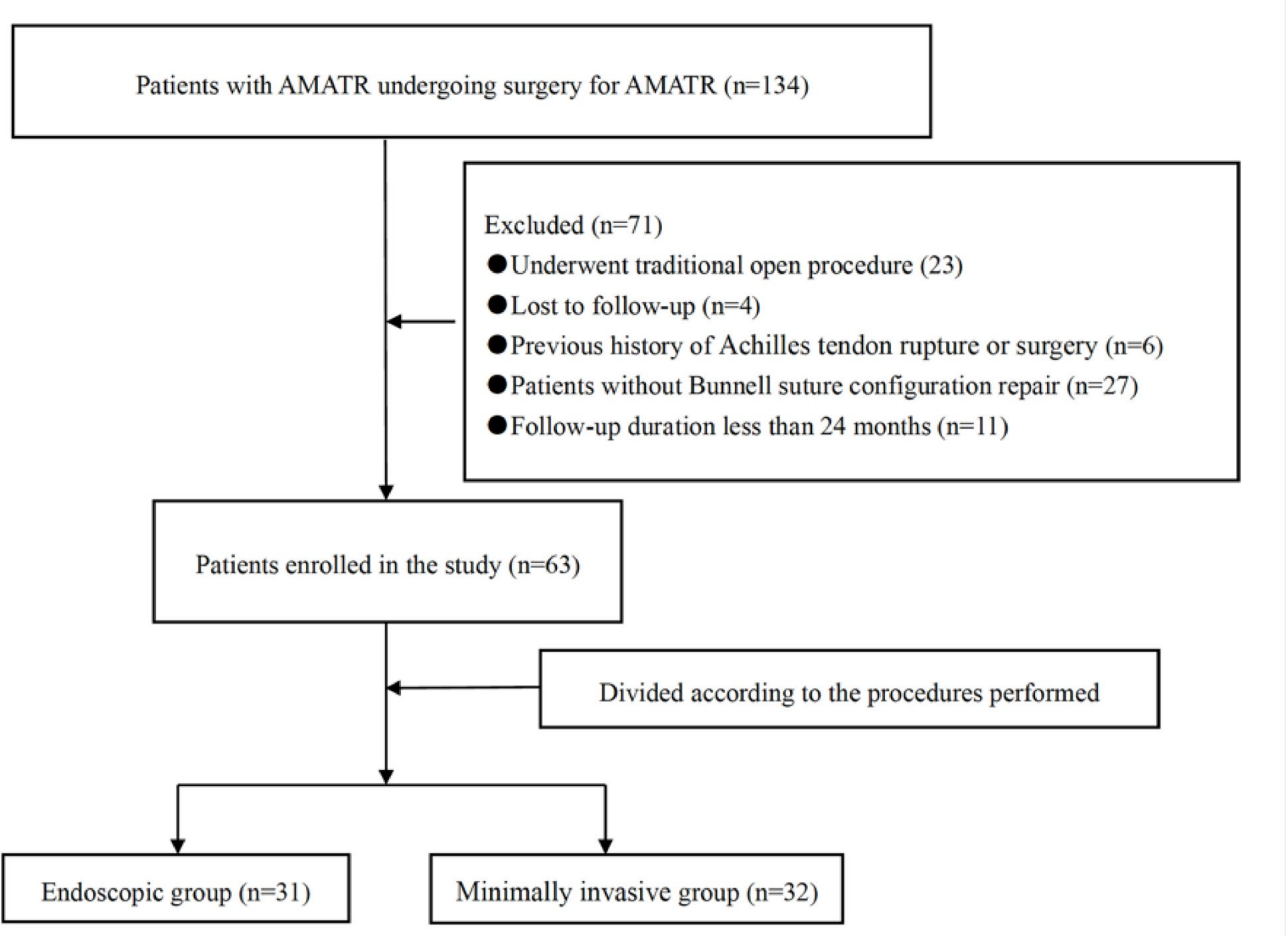
Study flow diagram of the study. AMATR, acute midsubstance Achilles tendon ruptures

A total of 63 patients with AMATR who fulfilled the inclusion and exclusion criteria were enrolled and treated at Xuzhou Clinical College of Xuzhou Medical University during the study period (Fig. 3). The study population consisted of 54 males and 9 females, with a mean age of 39.84 ± 10.40 years (range, 21–62 years). Ultimately, 31 patients underwent endoscopic repair (endoscopic group) and 32 underwent minimally invasive surgery (minimally invasive group). Demographic and injury-related variables, including age, sex, affected side, injury-to-surgery interval, and body mass index, were recorded. No significant baseline differences were observed between the two groups (Table 1).

At the 6-, 12-, and 24-month follow-up appointments, both groups demonstrated significant improvements in AOFAS and ATRS scores over time, with no significant between-group differences. Regarding secondary endpoints, the endoscopic group had a significantly longer operative time but a significantly shorter incision length compared with the minimally invasive group (*p* < 0.05). On postoperative days 1 and 3, VAS pain scores were significantly lower in the endoscopic group (*p* < 0.05). Patients in the endoscopic group returned to work significantly earlier (*p* < 0.05), while the time to return to sports was comparable between groups (*p* > 0.05) (Table 2).

No infections were observed in the endoscopic group, whereas three patients in the minimally invasive group developed superficial infection, which resolved with local wound care. The difference was not statistically significant (*p* > 0.05). Repeated-measures ANOVA revealed significant time effects for both AOFAS (F = 666.243, *p* < 0.001) and ATRS scores (F = 2539.546, *p* < 0.001), indicating progressive postoperative improvement. However, no significant group-time interactions were found, and overall score comparisons showed no significant differences between groups (*p* > 0.05), suggesting comparable long-term functional outcomes (Table 3).

**Table 1:** Characteristics of the two groups^a^

Variable	Endoscopic group (*n* = 31)	Minimally invasive group (*n* = 32)	p*-Value
Age, year	40.45 ± 10.90	39.25 ± 10.04	0.650‡
Sex			0.281†
Male	25(80.65%)	29(90.63%)	
Female	6(19.35%)	3(9.37%)	
BMI, Kg/m^2^	22.96 ± 2.97	22.67 ± 3.27	0.715‡
Injured side			0.721†
Left	10	9	
Right	21	23	
Injury-to-surgery interval, days	4.90 ± 2.44	5.22 ± 2.25	0.596†

BMI body mass index

^a^Data are presented as mean ± SD or No. (%)

*A value *p* < 0.05 was set as statistically significant

†Pearson χ^2^ test

‡t test

**Table 2:** General postoperative outcomes^a^

Variable	Endoscopic group (*n* = 31)	Minimally invasive group (*n* = 32)	P* Value
OT, min	40.90 ± 4.41	28.03 ± 4.37	<0.001‡
VAS			
day 1	5.10 ± 0.65	6.25 ± 0.80	<0.001‡
day 3	3.68 ± 0.75	4.63 ± 0.94	<0.001‡
IL, cm	1.63 ± 0.41	3.45 ± 0.41	<0.001‡
RTW, week	11.42 ± 1.03	12.63 ± 1.54	<0.001‡
RTS, week	22.03 ± 4.98	22.56 ± 1.74	0.573‡

OT, Operative time; VAS, Visual analogue scale; IL, Incision length; RTW, return to work; RTS, return to sports

^a^Data are presented as mean ± SD or No. (%)

*A value *p* < 0.05 was set as statistically significant

‡ t test

**Table 3:** Results from repeated-measures ANOVA on clinical scores ^a^

Variable	Endoscopic group (*n* = 31)	Minimally invasive group (*n* = 32)	Time effect (p*-value)	Group effect (p*-value)	Interaction effect (p*-value)	
AOFAS			<0.001†	0.646†	0.682†	
6 months	79.10 ± 2.36	78.81 ± 2.06				
12 months	90.35 ± 2.51	90.00 ± 3.33				
24 months	90.77 ± 2.45	90.59 ± 2.97				
ATRS			<0.001†	0.720†	0.111†	
6 months	85.29 ± 1.58	85.53 ± 1.76				
12 months	95.42 ± 1.95	95.00 ± 1.97				
24 months	95.74 ± 1.73	95.47 ± 1.72				

AOFAS, American orthopaedic foot and ankle society ankle-hindfoot score; ATRS, Achilles tendon total rupture score

^a^Data are presented as mean ± SD

*A value *p* < 0.05 was set as statistically significant

†A repeated measures analysis of variance (ANOVA)

## Discussion

The most important finding of the present study was that both the all-inside endoscopic repair and the minimally invasive modified Bunnell configuration provided dependable repair and yielded significant functional improvement in patients with AMATR, with comparable outcomes at 2-year follow-up. Although the all-inside endoscopic procedure achieved a smaller incision and enabled patients to return to work approximately one week earlier, the actual clinical relevance of these advantages appears limited. Moreover, compared with the minimally invasive procedure, the endoscopic approach requires radiofrequency equipment, which may increase overall treatment costs and thereby impose an additional burden on patients, although treatment expenses were not formally analysed in this study.

In the surgical management of AMATR, the choice of suture configuration is essential because it directly influences repair strength, tendon apposition, re-rupture risk, and postoperative recovery [22–24]. Conventional approaches have primarily focused on open repair methods [25–27], with widely used techniques including Krackow, Kessler, and Bunnell suture configurations. Each of these techniques presents distinct biomechanical and clinical advantages but also carries inherent limitations. The Krackow technique, with its characteristic locking-loop configuration, provides superior tensile strength and has become one of the most frequently adopted configurations in Achilles tendon repair; however, it necessitates a larger incision and is associated with a higher risk of infection and adhesion formation [28–29]. The Kessler technique, although simple and time-efficient, offers relatively lower mechanical resistance and is therefore less suitable for the high tensile demands of early postoperative mobilisation [30]. Although previous studies have reported that positioning the knot within the defect between the ruptured tendon ends may interfere with tendon healing and impair gliding function, the Bunnell configuration offers reliable mechanical stability [31]. To mitigate this issue, the endoscopic technique used in our study incorporated a modification in which the knot was relocated to the proximal tendon, outside the rupture site. This adjustment is intended to minimise mechanical irritation at the repair interface and create a more favourable environment for biological regeneration. In contrast, the minimally invasive procedure employed a traditional approach with the knot placed between the tendon stumps. Despite these technical differences, our findings demonstrated no significant differences in clinical outcomes or complication rates between the two groups. These results suggest that both the all-inside endoscopic modified Bunnell technique and the conventional minimally invasive method are reliable options for Achilles tendon repair, both achieving satisfactory healing and functional recovery.

In Achilles tendon repair, the incidence of wound-related complications is a critical determinant of postoperative recovery and patient satisfaction. In open surgical procedures, delayed wound healing (28.6%) and infection (20.9%) were reported in a cohort of 105 patients [32]. Yan et al. [33] documented an infection rate of 11.5% (9 of 80 patients) following channel-assisted minimally invasive repair. Even with percutaneous repair in cases of neglected Achilles tendon ruptures, the infection rate remained as high as 8.3% [34]. In the present study, although three cases of superficial infection were recorded in the minimally invasive group, the difference in complication rates between the two techniques was not statistically significant. Previous research has suggested that reduced disruption of the paratenon and adjacent soft tissues may preserve local vascularity, promote early tendon healing, and contribute to lower early postoperative pain [19, 35–37]. In both groups, meticulous protection of the paratenon was prioritised, and the paratenon was routinely repaired at the end of the minimally invasive procedure. This consistent preservation of the paratenon across surgical approaches may partially explain the favourable functional outcomes observed in both cohorts.

Functional evaluations demonstrated significant postoperative improvement in both AOFAS and ATRS scores over time, indicating effective restoration of tendon function regardless of the technique used. Repeated-measures ANOVA showed no group-time interaction, and final scores were comparable between the two groups. Therefore, endoscopic repair offers early benefits, but the long-term functional recovery trajectory is similar between endoscopic and minimally invasive techniques. The limited statistical power (< 10%) to detect small between-group differences further supports the interpretation that the two methods yield equivalent long-term outcomes.

For acute closed Achilles tendon ruptures, the prerequisite for all-inside endoscopic repair and the minimally invasive repair is a single-site rupture of the main body of the tendon, as multiple rupture sites demand greater suture strength and quality to achieve reliable fixation. Moreover, tears occurring at the musculotendinous junction are particularly challenging given the relatively loose tissue structure in this region; therefore, Krackow suturing or augmented suture techniques are recommended for repair [38]. In contrast, insertional ruptures of the Achilles tendon generally require tendon fixation combined with reconstruction of the insertion site [39].

Although statistically significant, the differences in VAS scores and return-to-work time seem clinically negligible, given the comparable functional outcomes across all postoperative time points. The earlier return to work observed in the arthroscopic group compared with the minimally invasive group may be attributed to greater confidence gained by both surgeons and patients when undergoing endoscopic procedures, which facilitates earlier functional activity. However, the difference was only one week, and unlikely to have any clinical relevance.

The present study has several noteworthy strengths. To our knowledge, this is the first study to describe the two-portal all-inside endoscopic modified Bunnell configuration technique for AMATR and to compare its clinical outcomes with those of minimally invasive repair. Second, all patients presented with isolated AMATR without any concomitant injuries, thereby reducing the potential influence of other factors on surgical outcomes. Third, all operations were conducted by a senior foot and ankle surgeon with specialised expertise in arthroscopic repair and minimally invasive techniques.

Despite these notable advantages, several limitations warrant consideration. First, this study was retrospective, and potential selection bias cannot be fully eliminated. Second, the sample size was limited and underpowered to detect subtle differences in long-term functional outcomes. Third, although no deep infections or re-ruptures occurred, the relatively small cohort limits broader conclusions regarding less common complications. Furthermore, the longer operative time associated with the endoscopic technique reflects the technical demands of the procedure, which requires advanced endoscopic skills and carries a learning curve. Patients’ subjective preferences were not formally assessed or quantified, which may have introduced unmeasured preference-related bias. Preoperative functional scores were not available, given the acute nature of Achilles tendon rupture, and long-term VAS scores were not collected. However, pain-related outcomes at later follow-up were indirectly captured through validated patient-reported outcome measures. The absence of preoperative baseline functional scores precluded formal analysis of the Minimum Clinically Important Difference based on change scores. This limitation is inherent to studies involving acute Achilles tendon rupture and should be considered when interpreting the functional outcomes. Additionally, although not directly evaluated, the increased cost of specialised equipment, such as endoscopes and radiofrequency devices, may affect the procedure’s overall cost-effectiveness.

## Conclusions

In summary, both the all-inside endoscopic and the minimally invasive modified Bunnell suture configurations provide reliable repair for AMATR and support a successful return to occupational and athletic activity. Although the all-inside endoscopic procedure was associated with longer operative time, it offered advantages of reduced early postoperative pain, smaller incisions, and earlier return to work without compromising functional recovery at the 2-year follow-up.

## Data Availability

The data from which this study arose are available from the corresponding authors upon reasonable request.

## Abbreviations

AMATR: Acute midsubstance Achilles tendon ruptures
VAS: Visual analog scale
AOFAS: American orthopaedic foot and ankle society score
ATRS: Achilles tendon total rupture score
BMI: Body mass index
MRI: Magnetic resonance imaging
